# Clinical outcomes of transcatheter edge-to-edge repair in patients with acute mitral regurgitation complicated by cardiogenic shock: a systematic review and meta-analysis

**DOI:** 10.1186/s12872-025-04844-z

**Published:** 2025-05-19

**Authors:** Ashraf Ahmed, Rafael Contreras, Anoop Gurram, Parsa Saberian, Rasha Kaddoura, Kayla Boyea, Yussif Issaka, Seyyed Mohammad Hashemi, Daniyal Ameen, Sara Nobakht, Ehsan Amini-Salehi, Sandeep Samethadka Nayak

**Affiliations:** 1 Department of Internal Medicine, Yale New Haven Health Bridgeport Hospital, Bridgeport, CT, USA; 2 Department of Hospital Medicine, Cleveland Clinic, 33300 Cleveland Clinic Blvd, Avon, OH 44011 USA; 3 Cardiovascular Research Center, Hormozgan University of Medical Sciences, Bandar Abbas, Iran; 4 Pharmacy Department, Heart Hospital, Hamad Medical Corporation, Doha, Qatar; 5 School of Medicine, Guilan University of Medical Sciences, Rasht, Iran

**Keywords:** Acute mitral regurgitation, Cardiogenic shock, Meta-analysis, MitraClip, Systematic review, Transcatheter edge-to-edge repair

## Abstract

**Background:**

Acute mitral regurgitation (AMR) complicated by cardiogenic shock (CS) is a critical cardiovascular emergency associated with high morbidity and mortality. Surgical intervention is often not feasible due to the unstable clinical status of these patients. Transcatheter edge-to-edge repair (TEER) has emerged as a minimally invasive alternative, yet its safety and efficacy in this specific population remain uncertain. This study aimed to systematically evaluate and synthesize the evidence on the clinical outcomes of TEER in patients with AMR complicated by CS.

**Methods:**

Databases including PubMed, Embase, and Web of Science were searched through March 4, 2025. Eligible studies included adult patients with AMR and CS undergoing TEER and reporting clinical outcomes. Data were synthesized using a random-effects model.

**Results:**

The pooled in-hospital mortality rate following TEER was 17.8% (95% CI: 11.2–25.2%). One-month mortality was 7.9% (95% CI: 1.1–16.8%), six-month mortality was 21.0% (95% CI: 11.2–32.7%), and one-year mortality was 36.5% (95% CI: 34.9–38.2%). Among patients with degenerative MR, the one-year mortality was 7.9% (95% CI: 0.8–19.0%), while for functional MR it was 9.4% (95% CI: 1.3–21.5%). Postprocedural MR reduction to ≤ grade 2 was achieved in 86.2% of patients (95% CI: 70.7–97.3%). The intra-aortic balloon pump (IABP) application rate was 57.9% (95% CI: 24.2%–88.5%). Compared to usual care, TEER significantly reduced in-hospital mortality (OR = 0.64; 95% CI: 0.51–0.81; *P* < 0.01). However, no significant reduction was found in rehospitalization risk (OR = 0.65; 95% CI: 0.14–3.03; *P* = 0.59).

**Conclusion:**

TEER appears to be a promising therapeutic option for patients with AMR complicated by CS. Compared to usual care, it is associated with significantly lower in-hospital mortality. However, high heterogeneity and low certainty of evidence highlight the need for further high-quality prospective studies to validate long-term outcomes and optimize patient selection.

**Clinical trial number:**

Not applicable.

**Graphical Abstract:**

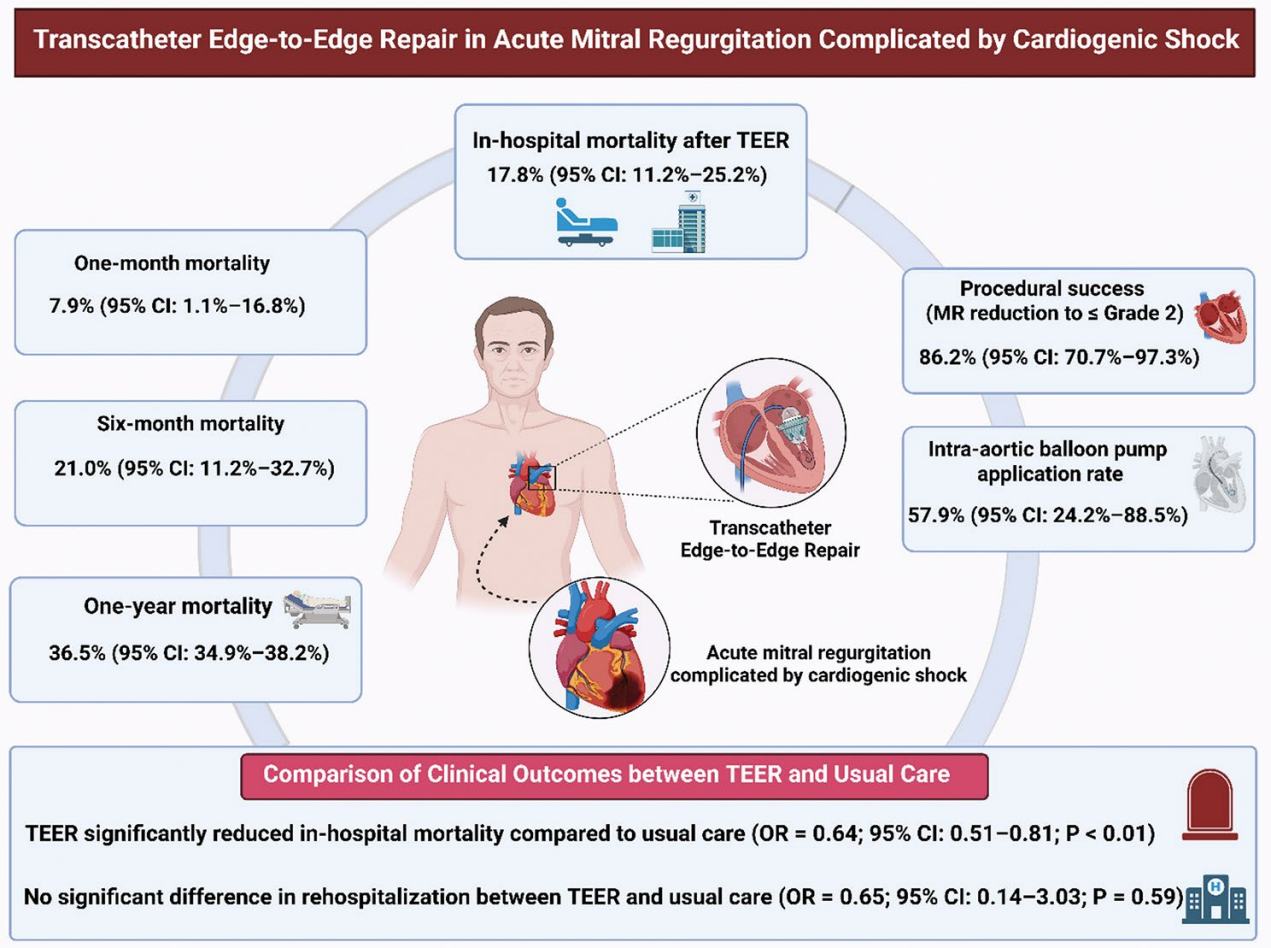

**Supplementary Information:**

The online version contains supplementary material available at 10.1186/s12872-025-04844-z

## Introduction

AMR is a life-threatening condition that often arises suddenly, typically due to ischemic papillary muscle rupture, infective endocarditis, or spontaneous chordae tendineae rupture [1–4]. When AMR occurs, it can rapidly lead to volume overload of the left atrium and ventricle, resulting in pulmonary edema and hemodynamic instability [5–7]. In severe cases, this cascade may culminate in CS, a critical state marked by inadequate tissue perfusion and end-organ dysfunction [8, 9]. The combination of AMR and CS represents a complex clinical challenge with high morbidity and mortality, demanding prompt recognition and effective intervention [8, 10].

Surgical mitral valve repair or replacement has traditionally been the standard of care for severe AMR [11, 12]. However, the hemodynamic fragility of patients with concurrent CS often makes them poor surgical candidates due to prohibitive perioperative risk [13]. In recent years, TEER, most commonly performed using the MitraClip system, has emerged as a less invasive alternative to surgical intervention [14–16]. Initially approved for chronic mitral regurgitation, particularly in patients with functional or degenerative etiology, TEER has seen expanding use in acute and emergent scenarios, including in the setting of AMR complicated by CS [17–22].

Early observational studies and case series suggest that TEER may offer hemodynamic stabilization, improve mitral valve competence, and potentially reduce short-term mortality in this critically ill population. However, data on its safety, efficacy, and long-term outcomes remain limited and scattered across small, heterogeneous studies [23–25]. Given the urgent nature of these clinical situations, robust evidence is necessary to guide decision-making and optimize patient outcomes.

This systematic review and meta-analysis aims to synthesize the current body of evidence evaluating the use of TEER in patients with AMR complicated by CS. By consolidating existing data, we seek to provide a clearer understanding of the role of TEER in this high-risk group and identify gaps that warrant further investigation.

## Methods

This meta-analysis was conducted in accordance with the methodological standards outlined in the Cochrane Handbook for Systematic Reviews and reported following the Preferred Reporting Items for Systematic Reviews and Meta-Analyses (PRISMA) guidelines [26, 27]. The review protocol was prospectively registered in the PROSPERO database (Registration ID: CRD42023411997).

### Search strategy

We conducted a comprehensive literature search across PubMed, Embase, and Web of Science from inception up to March 4, 2025. The search strategy combined terms related to “acute mitral regurgitation,” “cardiogenic shock,” and “transcatheter edge-to-edge repair” (e.g., MitraClip). Keywords and MeSH terms were adapted for each database. Reference lists of relevant studies and reviews were also manually screened to identify additional eligible articles. Detailed search formula for each database is presented in table S1

## Study selection and eligibility criteria

Studies were eligible for inclusion if they met the following criteria: (1) involved adult patients diagnosed with AMR complicated by CS (2), evaluated outcomes following TEER, and (3) reported clinical endpoints such as procedural success, in-hospital or short-term mortality, or hemodynamic outcomes. Single case reports, review articles, editorials, and studies lacking sufficient outcome data were excluded. Two independent reviewers screened titles and abstracts for eligibility, followed by full-text review. Discrepancies were resolved through consensus or adjudication by a third reviewer.

## Quality assessment

To evaluate the methodological quality of each study included in the review, we employed the Joanna Briggs Institute (JBI) critical appraisal checklists tailored to the relevant study designs (e.g., cross-sectional, cohort) [28–30]. Discrepancies in the quality assessments were addressed through discussion or by seeking input from a third reviewer.

## Data extraction

A standardized data extraction form was used to collect information on study characteristics (e.g., design, sample size, setting), patient demographics, etiology of AMR, procedural details, and clinical outcomes. Data were independently extracted by two reviewers and cross-verified for accuracy. When necessary, study authors were contacted for clarification or additional data.

### Statistical analysis

For the statistical analysis, a random-effects model was employed using Restricted Maximum Likelihood (REML) estimation to account for expected between-study variability. Heterogeneity among studies was assessed using I², as well as Cochran’s Q test. An I² value exceeding 50% in conjunction with a Q test p-value less than 0.10 was considered indicative of substantial heterogeneity. Outlier detection was performed using Galbraith plots, which allowed identification of studies exerting disproportionate influence on overall heterogeneity. To evaluate potential publication bias, we utilized Begg’s and Egger’s tests, along with the trim-and-fill method, ensuring a comprehensive assessment of bias within the included studies. STATA version 18 was used for the analysis.

## Results

### Study selection

**Fig. 1:**
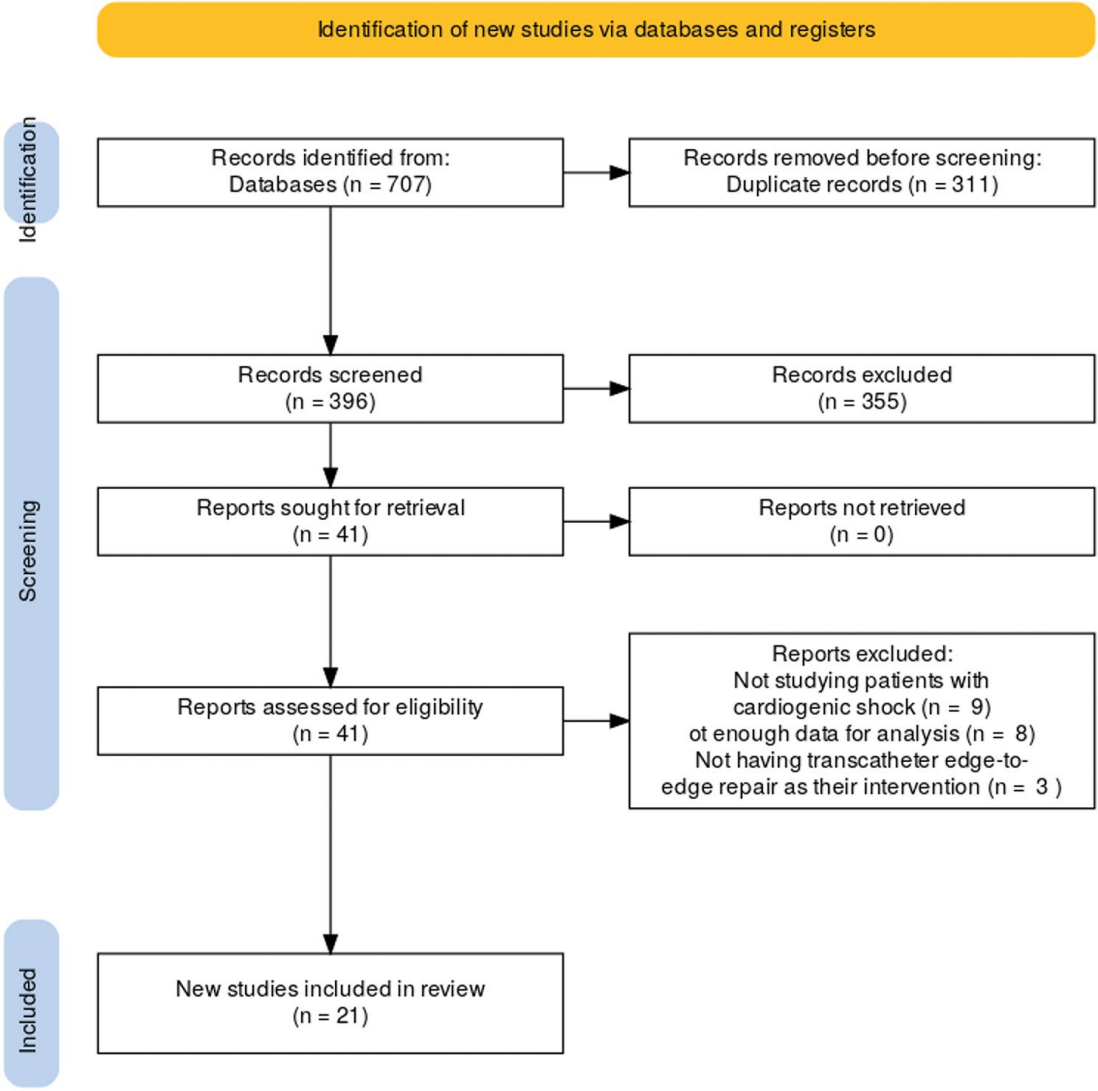
Study selection process

A total of 707 records were identified through database searches. After removing 311 duplicate records, 396 records were screened for eligibility. Of these, 355 records were excluded based on their titles and abstracts, as they did not meet the inclusion criteria. Subsequently, 41 reports were sought for full-text retrieval, and all of these reports were successfully retrieved. Upon full-text review, 41 reports were assessed for eligibility. Of these, 20 reports were excluded because they did not meet the inclusion criteria or lacked sufficient information for analysis. A total of 21 studies were included in the final review (Fig. 1).

## Study characteristics

This systematic review and meta-analysis incorporated 21 studies [21, 31–50] published between 2017 and 2024, encompassing a total of 622 patients with AMR complicated by CS who underwent transcatheter mitral valve repair. The majority of studies were retrospective cohort analyses, with a smaller number comprising case series. Geographically, the included studies spanned North America, Europe, Asia, and the Middle East, reflecting a diverse international experience.

The mean age of patients in the CS subgroup ranged from 57 to 81.7 years across studies, with considerable variation in gender distribution. Several cohorts reported a predominance of male patients, including Perel et al. [44], in which 92% of the cohort were men​. Surgical risk stratification, when reported, utilized the Society of Thoracic Surgeons (STS) score or EuroSCORE II, both of which consistently indicated high operative risk.

Hospital length of stay among CS patients ranged from approximately 10 to 38 days. Procedural success—most commonly defined as postprocedural mitral regurgitation reduction to ≤ grade 2—was achieved in over 80% of cases in most cohorts. However, reporting of key clinical variables such as hypertension (HTN), coronary artery disease (CAD), left ventricular ejection fraction (LVEF), and mean pulmonary artery pressure (mPAP) was inconsistent across studies. The quality of included studies is provided in table S2 (Table 1).

**Table 1:** Study characteristics

First author	Year of publication	Time set	Study Design	Mean Age CS group	Gender Distribution (M/F)	The country	Population TMVR (CS/without CS)	Population CS group	STS score CS group	hospital length of stay Mean (SD), day	postprocedural MR ≤ 2+	HTN CS group	CAD CS Group	LVEF CS group	mPAP CS group
P. Kovach et al. [41]	2021	January 2018 and March 2019	Retrospective Cohort	72 ± 13	(10/10)	USA	20(8/12)	8	N. A	N. A	N. A	N. A	N. A	N. A	N. A
Lee et al. [42]	2021	2019–2021	Cohort	72.6 ± 18.2	(6/34)	Taiwan	50(8/42)	8	19.7 ± 12.0	N. A	7	6	6	40.4 ± 16.8	40.6 ± 10.5
Simard et al. [46]	2022	November 2013 to December 2021	Retrospective Cohort	73.0 ± 11.9	(1519/2278)	USA	3797 (3797/0)	3797	14.9 ± 15.3	12.5 ± 15.0	3397	3132	1488	41.1 ± 17.5	N. A
Haberman et al. [39]	2024	December 2009 to September 2022	Retrospective Cohort	68 ± 14	(10/13)	North America & Europe and Middle East	23(20/3)	20	N. A	11.3 ± 7.98	13	N. A	15	45.6 ± 7.96	N. A
Jung et al. [40]	2021	January 2011 and February 2019	Retrospective Cohort	68.9 ± 12.1	(78/63)	North America & Europe	141(141/0)	141	16.1 ± 16.6	12.0 ± 10.49	N. A	85	86	33.8 ± 14.0%	36.9 ± 10.1
Tang et al. [49]	2021	January 2014 to March 2019	Retrospective Cohort	71 ± 11	(257/492)	USA	596(596/0)	596	N. A	17.0 ± 11.15	N. A	542	421	N. A	N. A
Adamo et al. [31]	2017	October 2010 to October 2015	Case series	73 ± 6	(2/3)	Italy	5(4/1)	4	N. A	N. A	4	N. A	N. A	N. A	N. A
Makmal et al. [43]	2024	July 2012 to March 2022	Retrospective cohort	73.5 ± 10.9	(10/21)	Isreal	31(9/22)	9	N. A	N. A	N. A	N. A	N. A	N. A	N. A
Aldrugh et al. [21]	2021	2010–2018	Retrospective cohort	73. ± 14	(243/364)	USA	607(607/0)	607	N. A	17.33 ± 12.63	N. A	497	419	N. A	N. A
Estévez-Loureiro et al. [35]	2021	January 2016 to February 2020	Retrospective cohort	68 ± 10	(48/45)	North America and Europe	93(50/43)	50	N. A	N. A	45	33	N. A	34 ± 12	40 ± 13
So et al. [47]	2022	Jan 2014 to December 2019	Retrospective cohort	70.3 ± 11.5	8(3/5)	USA	8(8/0)	8	31.0 ± 10.5	N. A	5	N. A	N. A	54.1 ± 8.4	N. A
Falasconi et al. [36]	2021	2012 to 2019	Retrospective cohort	70.3 ± 3.8	(4/27)	Italy and USA	31(31/0)	31	37.9 ± 8.89	N. A	27	N. A	24	30.0 ± 8.86	30.7 ± 5.67
Flint et al. [37]	2019	November 2013 to October 2018	Retrospective cohort	71.7 ± 12.8	(56/79)	USA	135(12/123)	12	33.4 ± 22.3	N. A	12	N. A	N. A	37 ± 15	38 ± 11/33
Perel et al. [44]	2022	January 2020 to July 2021	Retrospective cohort	70.3 ± 10.3	(1/12)	Isreal	13(13/0)	13	N. A	10.33 ± 13.29	13	N. A	N. A	N. A	N. A
Cheng et al. [33]	2019	January 2014 and August 2018	Retrospective cohort	65.5 ± 17.0	(5/24)	USA	29(29/0)	29	N. A	N. A	26	N. A	N. A	27.3 ± 16.6	N. A
Rizik et al. [45]	2019	2019	Case series	81.7 ± 10.0	(0/3)	USA	3(3/0)	3	N. A	N. A	3	N. A	N. A	N. A	N. A
Garcia et al. [38]	2020	2010–2019	Retrospective cohort	74 ± 11	(5/6)	USA	11(11/0)	11	N. A	N. A	8	N. A	N. A	N. A	N. A
Chitturi et al. [34]	2020	Early 2020	Case series	58.0 ± 2.8	(0/2)	USA	2(2/0)	2	N. A	N. A	2	N. A	N. A	N. A	N. A
Vandenbriele et al. [50]	2021	August 2017 and January 2020	Case series	66.8 ± 4.9	(2/4)	USA	6(6/0)	6	N. A	17.5 ± 5.2	6	N. A	N. A	N. A	N. A
Tanaka et al. [48]	2022	late 2020 and early 2022	Case series	69 ± 2.65	(1/2)	Japan	3(3/0)	3	N. A	N. A	3	N. A	N. A	N. A	N. A
Ahmed et al. [32]	2023	N.A	Case series	57 ± 7.1	(0/2)	Qatar	2(2/0)	2	N. A	N. A	2	N. A	N. A	N. A	N. A

Abbreviation: CS, Cardiogenic shock; TMVR, Transcatheter Mitral Valve Replacement; STS score, Society of Thoracic Surgeons risk score; HTN, Hypertension; CAD, Coronary artery disease; LVEF, Left ventricular ejection fraction; mPAP, mean pulmonary artery pressure

### Clinical outcome of TEER

#### In-hospital mortality rate

**Fig. 2:**
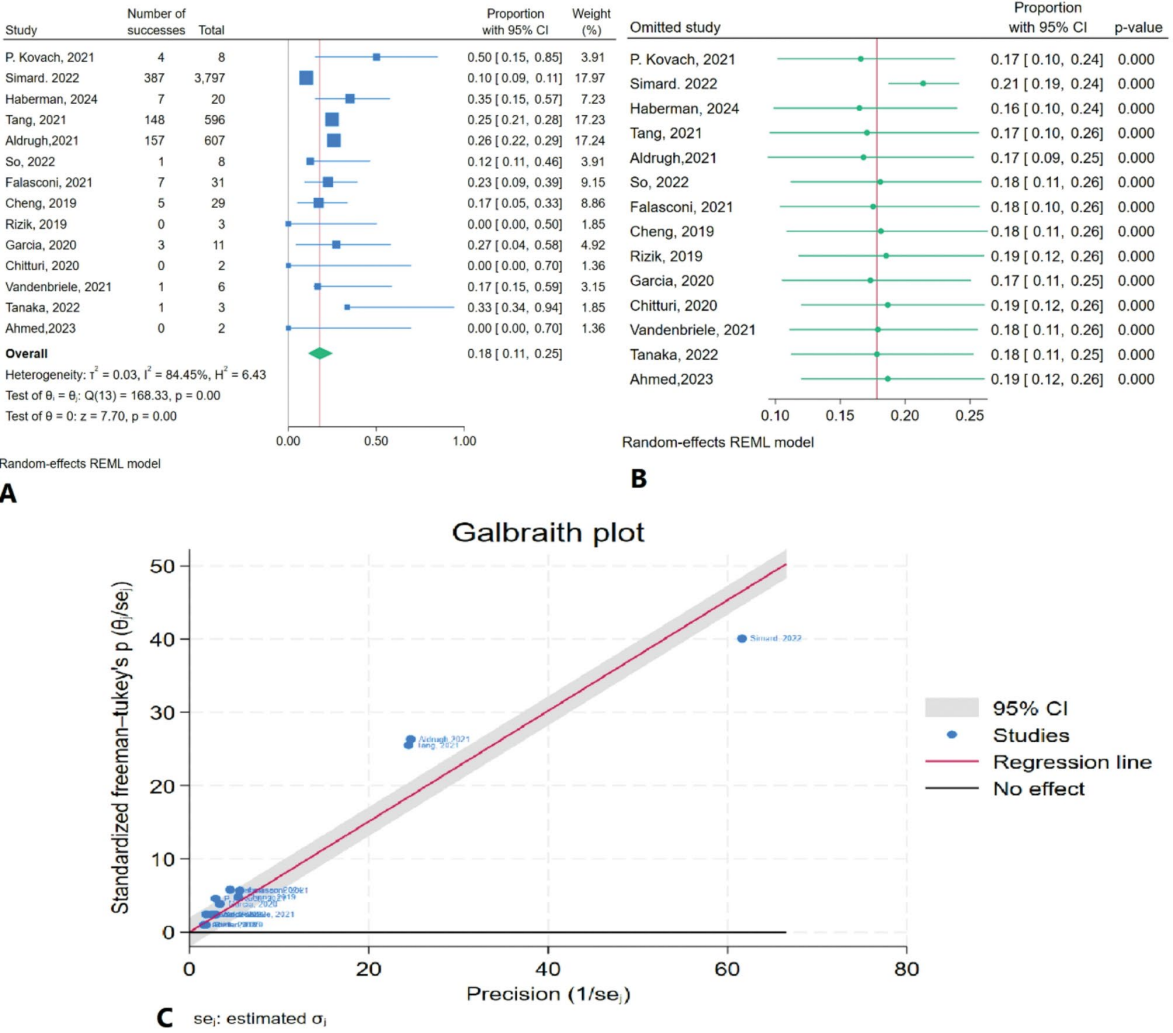
In-hospital mortality rate of TEER in patients with CS: **A**: Forest plot, **B**: Sensitivity analysis, **C**: Galbraith plot

The meta-analysis results indicated that the prevalence of in-hospital mortality was 17.8% (95% CI: 11.2–25.2%) with considerable heterogeneity (I² = 84.45%) (Fig. 2A). Sensitivity analysis demonstrated no substantial changes after the removal of each individual study (Fig. 2B). The Galbraith plot identified outliers, including Simard et al. (2022), Aldrugh et al. (2021), and Tang et al. (2021) (Fig. 2C).

### One month mortality rate

**Fig. 3:**
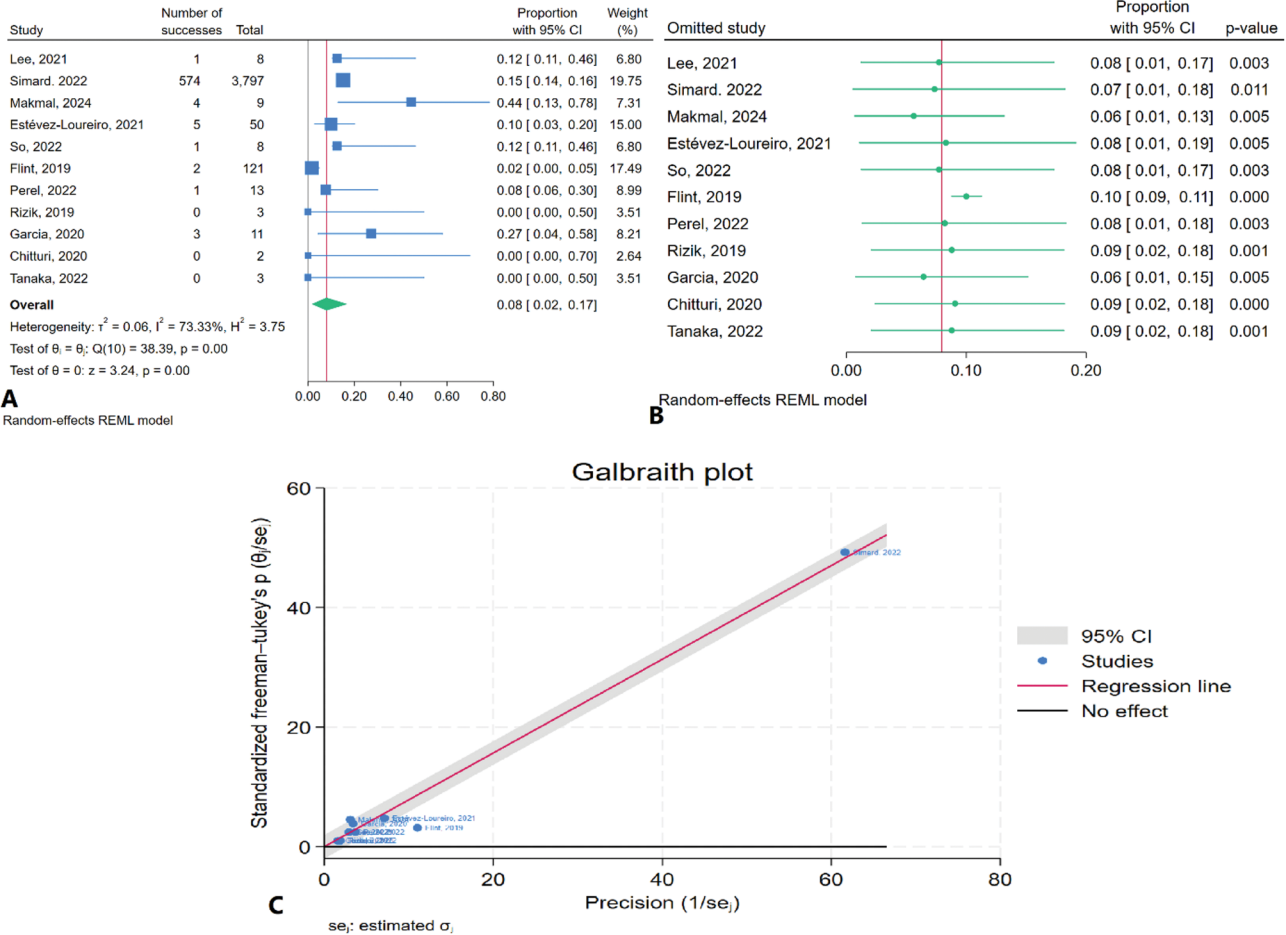
One-month mortality rate of TEER in patients with CS: **A**: Forest plot, **B**: Sensitivity analysis, **C**: Galbraith plot

The meta-analysis results indicated that the pooled one-month mortality rate was 7.9% (95% CI: 1.07–16.8%) with moderate heterogeneity (I² = 73.33%) (Fig. 3A). Sensitivity analysis demonstrated no substantial changes in the pooled estimate after the removal of any individual study, suggesting the robustness of the findings (Fig. 3B). The Galbraith plot did not identify any studies as outliers (Fig. 3C).

### Six-month mortality rate

**Fig. 4:**
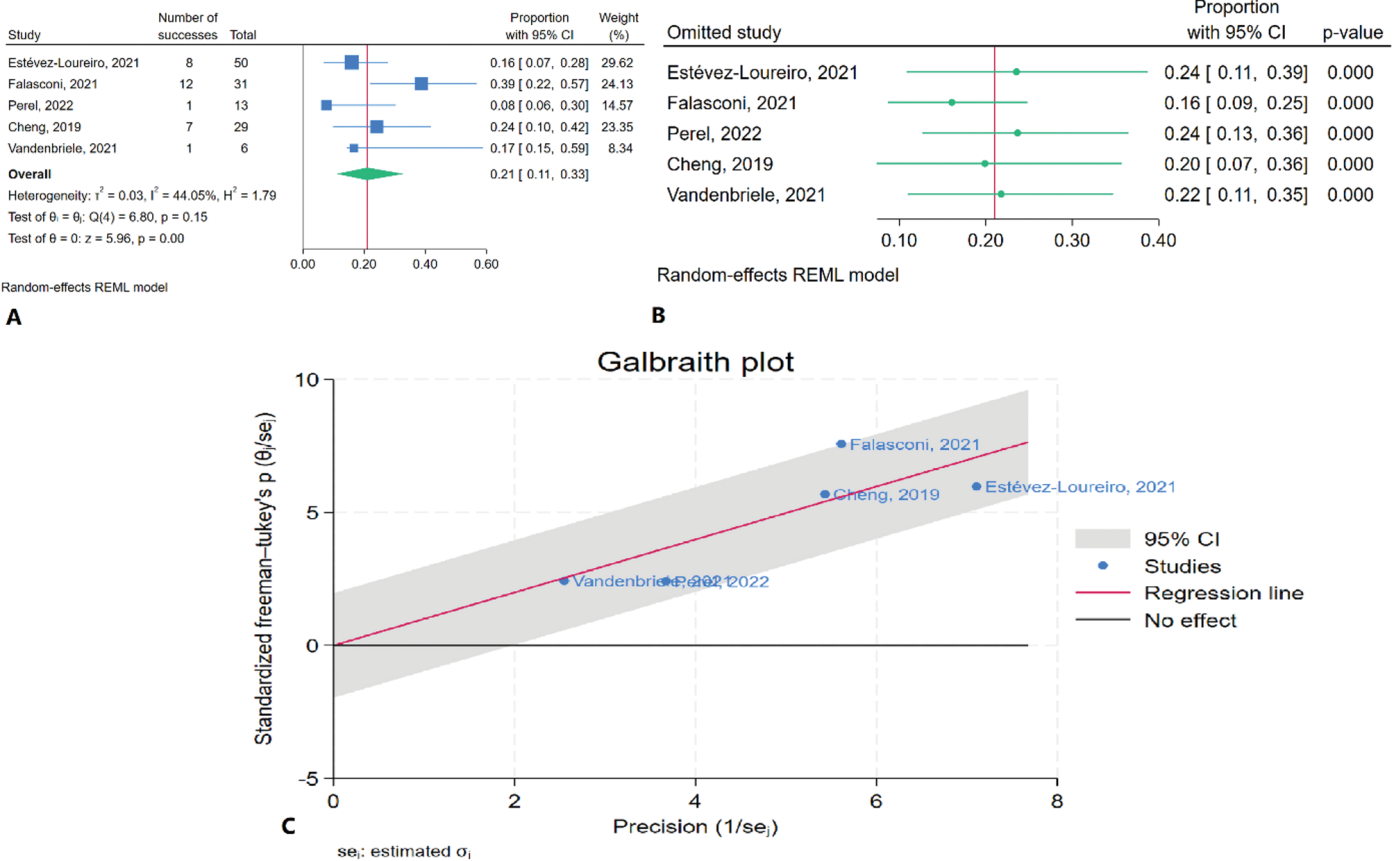
Six-month mortality rate of TEER in patients with CS: **A**: Forest plot, **B**: Sensitivity analysis, **C**: Galbraith plot

The meta-analysis results indicated that the pooled six-month mortality rate was 21.0% (95% CI: 11.2–32.7%) with non-significant heterogeneity (I² = 44.05%) (Fig. 4A). Sensitivity analysis showed no substantial changes in the pooled estimate after the removal of any individual study, suggesting that the findings were consistent across all studies (Fig. 4B). The Galbraith plot did not identify any studies as outliers (Fig. 4C).

### One-year mortality rate

**Fig. 5:**
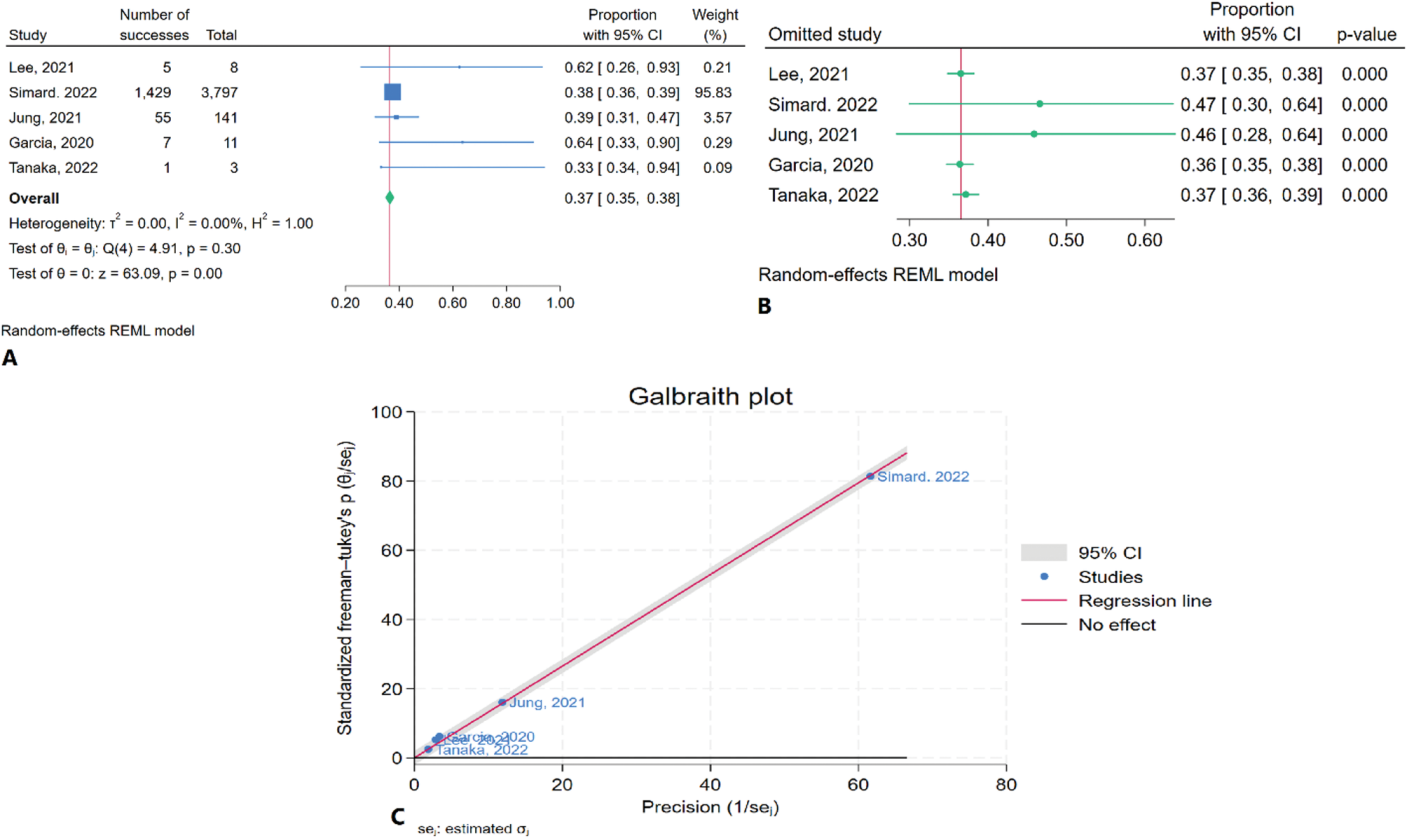
One-year mortality rate of TEER in patients with CS: **A**: Forest plot, **B**: Sensitivity analysis, **C**: Galbraith plot

The meta-analysis results indicated that the pooled one-year mortality rate was 36.5% (95% CI: 34.9–38.2%) with no heterogeneity (I² = 0.00%) (Fig. 5A). Sensitivity analysis showed no significant changes in the pooled estimate after the removal of any individual study (Fig. 5B). The Galbraith plot did not identify any studies as outliers (Fig. 5C).

### One-year mortality rate due to degenerative MR

**Fig. 6:**
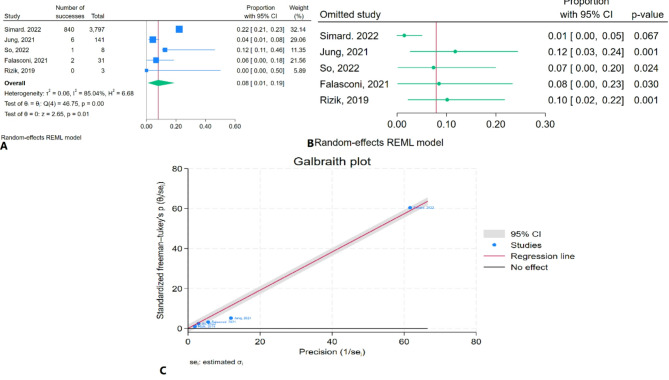
One-year mortality rate due to degenerative MR of TEER in patients with CS: **A**: Forest plot, **B**: Sensitivity analysis, **C**: Galbraith plot

The meta-analysis results indicated that the pooled one-year mortality rate due to degenerative MR was 7.9% (95% CI: 0.8–19.0%) with significant heterogeneity (I² = 85.04%) (Fig. 6A). Sensitivity analysis showed a significant change in the pooled estimate after the removal of Simard et al. (2022) (Fig. 6B). The Galbraith plot identified Jung et al. (2021) as outlier (Fig. 6C).

### One-year mortality rate due to functional MR

**Fig. 7:**
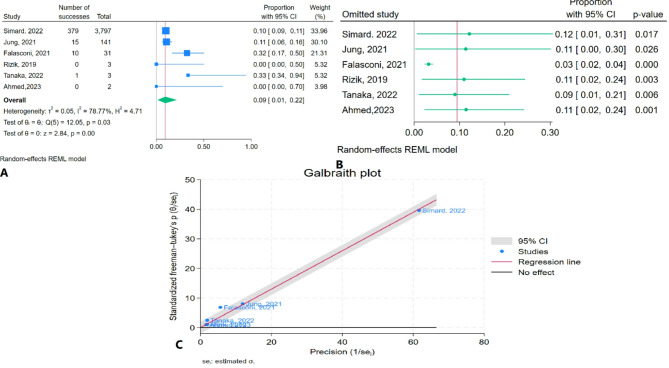
One-year mortality rate due to functional MR of TEER in patients with CS: **A**: Forest plot, **B**: Sensitivity analysis, **C**: Galbraith plot

The meta-analysis results indicated that the pooled one-year mortality rate due to functional MR was 9.4% (95% CI: 1.3–21.5%) with high heterogeneity (I² = 78.77%) (Fig. 7A). Sensitivity analysis showed a significant change in the pooled estimate after the removal of Falasconi et al. (2021) (Fig. 7B). The Galbraith plot identified Falasconi et al. (2021) as an outlier (Fig. 7C).

### Intra-aortic balloon pump (IABP) application rate

**Fig. 8:**
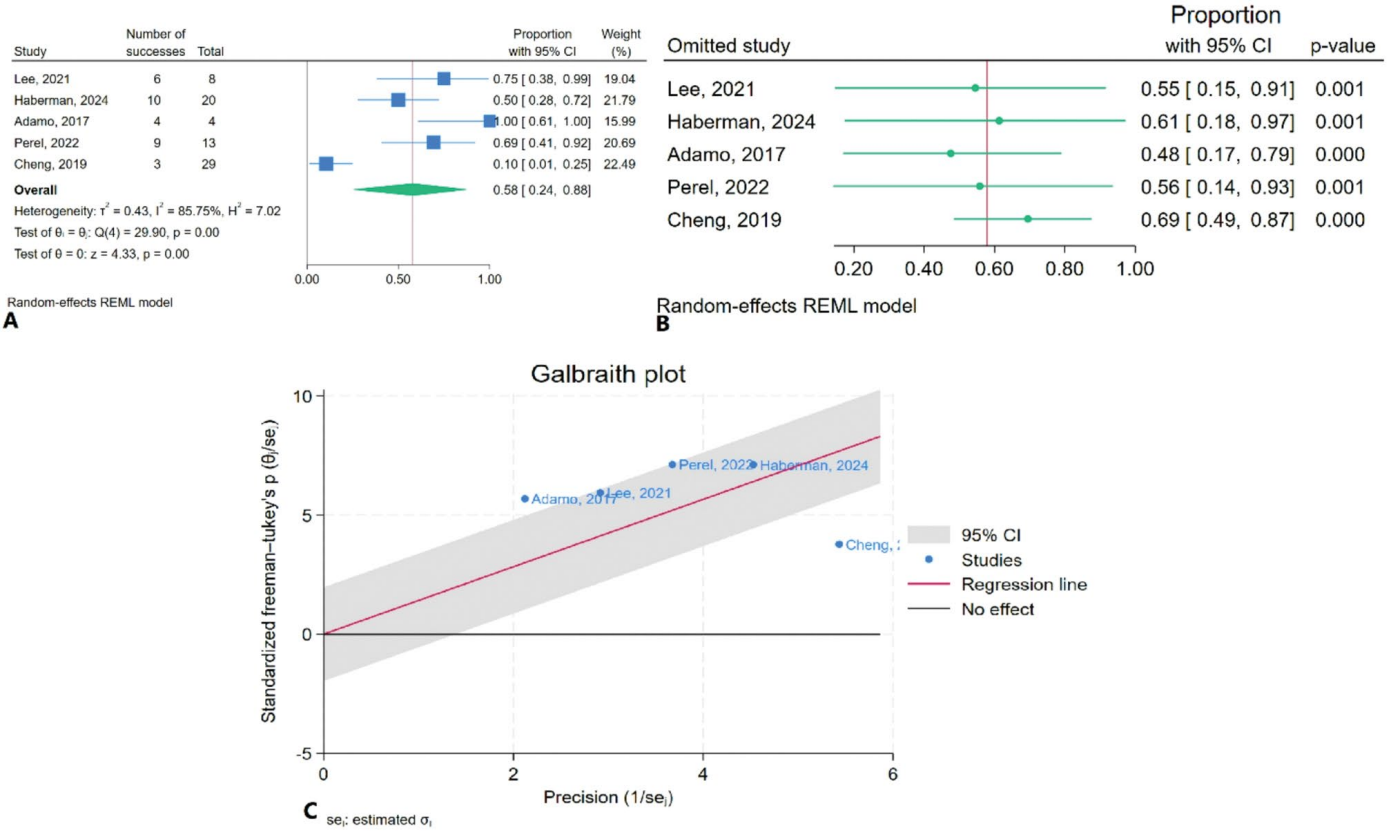
Intra-aortic balloon pump application rate after TEER in patients with CS: **A**: Forest plot, **B**: Sensitivity analysis, **C**: Galbraith plot

The meta-analysis results indicated that the pooled IABP application rate was 57.9% (95% CI: 24.2–88.5%) with high heterogeneity (I² = 85.75%) (Fig. 8A). Sensitivity analysis showed no substantial change in the pooled estimate after the removal of any individual study (Fig. 8B). The Galbraith plot identified Cheng et al. (2019) and Adamo (2017) as outliers (Fig. 8C).

### Postprocedural reduction in MR severity to ≤ grade 2

**Fig. 9:**
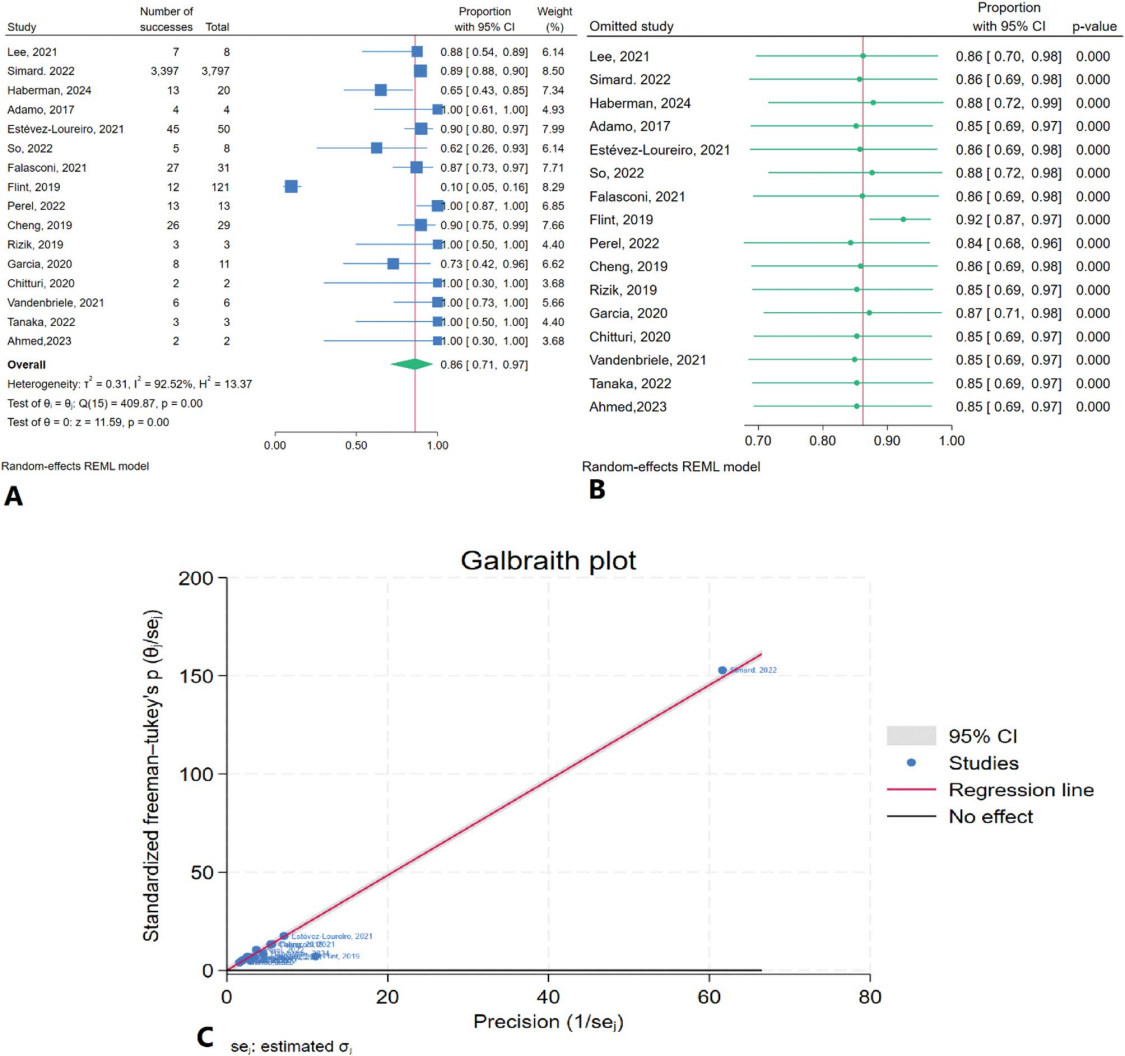
Intra-aortic balloon pump application rate after TEER in patients with CS: **A**: Forest plot, **B**: Sensitivity analysis, **C**: Galbraith plot

The meta-analysis results indicated that the pooled postprocedural reduction in MR severity to ≤ grade 2 was 86.2% (95% CI: 70.7–97.3%) with high heterogeneity (I² = 92.52%) (Fig. 9A). Sensitivity analysis showed no significant changes in the pooled estimate after the removal of any individual study (Fig. 9B). The Galbraith plot identified Simard et al. (2022) as an outlier (Fig. 9C).

### In-hospital mortality comparison of TEER versus usual care

**Fig. 10:**
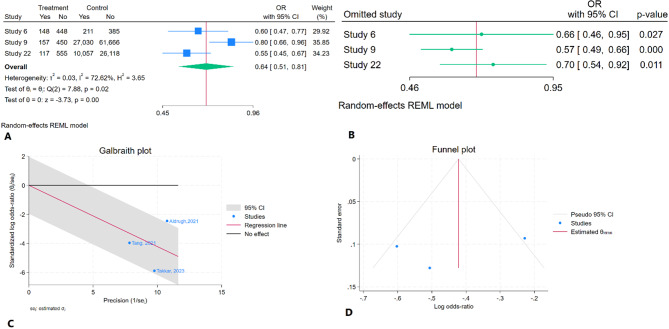
Results of meta-analysis for in-hospital mortality of TEER vs. usual care in patients with CS. **A**: Forest plot **B**: Sensitivity analysis **C**: Galbraith plot D: Trim and fill analysis

The meta-analysis demonstrated that TEER significantly reduces in-hospital mortality compared to usual care (OR = 0.64, 95% CI: 0.51–0.81, *P* < 0.01). Moderate heterogeneity was present (I² = 72.62%) (Fig. 10A). In the sensitivity analysis, removing individual studies did not significantly alter the overall effect (Fig. 10B). Aldrugh, 2021 was identified as an outlier in the Galbraith plot analysis (Fig. 10C). Begg’s test (*P* = 1) and Egger’s test (*P* = 0.54) showed the absence of significant publication bias. Additionally, trim-and-fill analysis suggested no missing studies (Fig. 10D). According to the GRADE criteria, the overall strength of the evidence was rated as very low (Table 2).

### Rehospitalization comparison of TEER versus usual care

**Fig. 11:**
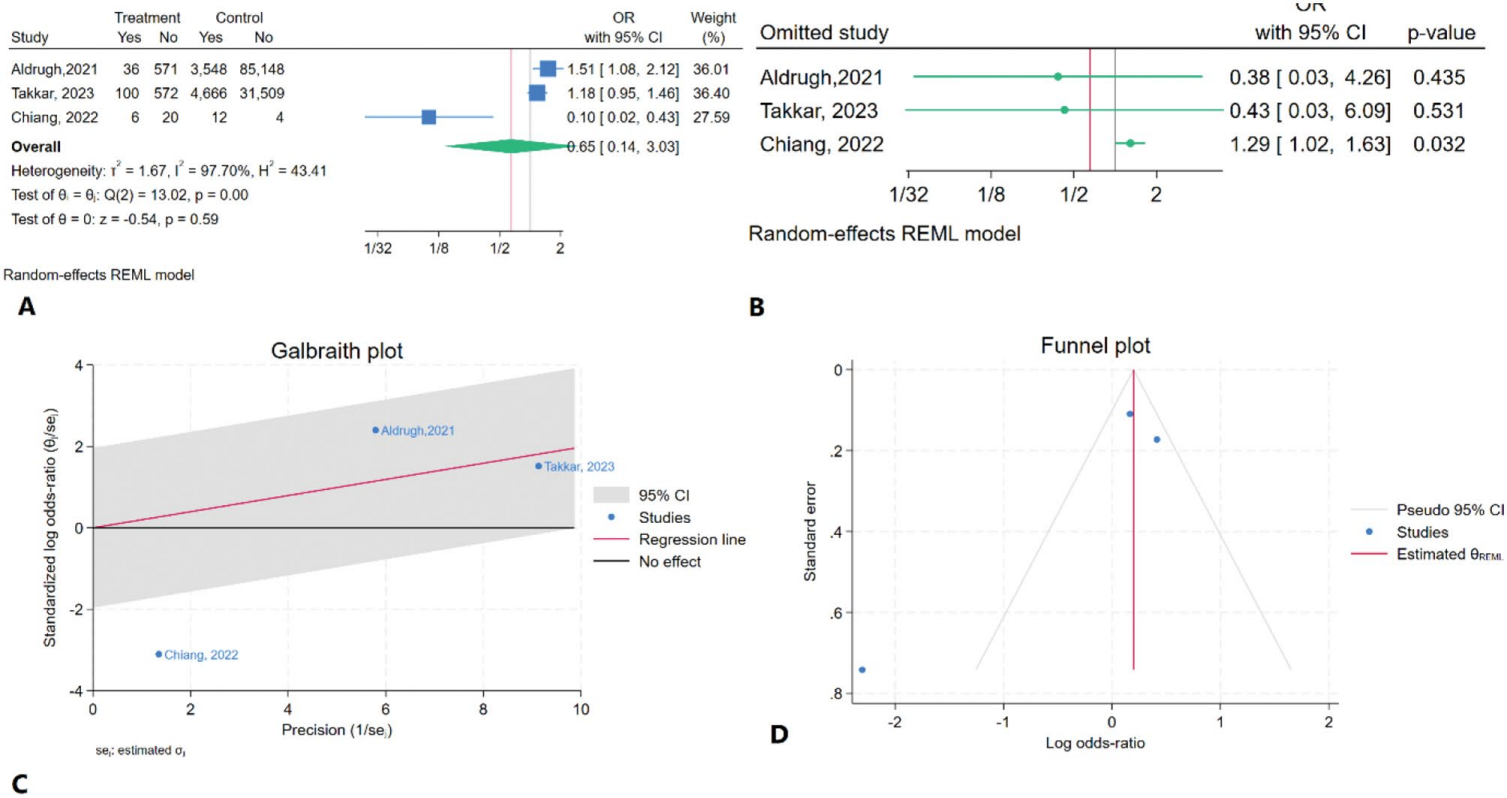
Results of meta-analysis for rehospitalization of TEER vs. usual care in patients with CS. **A**: Forest plot **B**: Sensitivity analysis **C**: Galbraith plot **D**: Trim and fill analysis

The meta-analysis demonstrated that TEER did not significantly reduce the risk of rehospitalization compared to usual care (OR = 0.65, 95% CI: 0.14–3.03, *P* = 0.59). Severe heterogeneity was observed (I² = 99.70%) (Fig. 11A). In the sensitivity analysis, removal of the study by Chiang (2022) rendered the results statistically significant (Fig. 11B). Chiang (2022) was also identified as an outlier in the Galbraith plot (Fig. 3C). Begg’s test (*P* = 1.00) indicated no significant publication bias, while Egger’s test suggested the presence of significant publication bias (*P* < 0.01). The trim-and-fill analysis did not impute any missing studies (Fig. 3D). According to the GRADE criteria, the overall certainty of the evidence was rated as very low (Table 2).

**Table 2:** Results of GRADE assessment

Quality assessment							Quality
No of studies	Design	Risk of bias	Inconsistency	Indirectness	Imprecision	Other considerations	
In-hospital mortality of TEER vs. usual care							
3	Observational studies	No serious risk of bias	Serious	No serious indirectness	No serious imprecision	None	Very low
**Rehospitalization of TEER vs. usual care**							
3	Observational studies	No serious risk of bias	Very serious	No serious indirectness	no serious imprecision	None	Very low

## Discussion

In this systematic review and meta-analysis, we comprehensively evaluated the clinical outcomes of TEER in patients with AMR complicated by CS—a population characterized by extreme hemodynamic instability and high procedural risk. The pooled in-hospital mortality rate following TEER was 17.8%, and postprocedural MR reduction to ≤ grade 2 was achieved in 86.2% of patients, reflecting favorable short-term procedural success. Notably, TEER was associated with a statistically significant reduction in in-hospital mortality compared to usual care (OR = 0.64; 95% CI: 0.51–0.81; *P* < 0.01), suggesting a potential survival benefit in patients traditionally considered poor surgical candidates. Additionally, IABP support was utilized in 57.9% of cases, indicating the frequent need for adjunctive mechanical circulatory support in this high-acuity setting. While the short-term outcomes are encouraging, the pooled one-year mortality rate remained high at 36.5%, underscoring the ongoing clinical vulnerability and complex pathophysiology associated with AMR and CS. Collectively, these findings highlight the clinical promise of TEER in stabilizing critically ill patients with AMR and CS.

Our findings align with and extend those reported by Saito et al. (2024), who conducted a meta-analysis of TEER in patients with CS and MR. While both studies found that TEER effectively reduced MR, with 88% of patients in Saito et al.’s study achieving MR severity of less than 2+, our study observed a similar outcome, with 86% of patients showing a reduction in MR severity to less than 2+. Additionally, while Saito et al. reported an in-hospital mortality rate of 11%, with 30-day and 1-year mortality rates of 15% and 36%, respectively, our study showed slightly higher mortality rates: 18% in-hospital, 8% at 30 days, 21% at 6 months, and 37% at 1 year. It is also worth noting that our study included a larger number of studies [51].

The findings of this meta-analysis are consistent with previous research evaluating the efficacy of TEER in patients with AMR complicated by CS. In a recently published comprehensive systematic review involving 727 patients, Dimitriadis et al. reported a 30-day mortality rate of 14.2% and MR reduction to ≤ grade 2 in 89.2% of cases, closely mirroring the results of our pooled analysis [52]. Similarly, Yokoyama et al. documented a pooled in-hospital mortality rate of 11.8% in hemodynamically unstable patients undergoing TEER, along with high procedural success rates and without major procedural complications [23]. In addition, Haberman et al. emphasized the expanding role of TEER in the management of both primary and secondary MR following acute myocardial infarction, particularly among patients with CS who are considered poor surgical candidates. Their synthesis of data from multiple case series and registries revealed procedural success rates exceeding 85%, accompanied by meaningful improvements in hemodynamic profiles and survival [53].

Further support for the clinical utility of TEER in high-risk populations is provided by studies such as those by Chiang et al. [54] and Perel et al. [44], both of which reinforce its value in patients with CS and significant mitral regurgitation. Chiang et al. demonstrated that TEER was associated with a significantly lower incidence of major adverse cardiovascular events at 30 days and six months compared to medical therapy alone, alongside fewer heart failure readmissions and improved clinical status [54]. Complementary findings by Perel et al. in a cohort of patients with refractory CS, largely due to ischemic mitral regurgitation, showed a 30-day survival rate of 92% and 100% six-month survival among initial survivors. Notably, MR reduction led to prompt hemodynamic stabilization, with over half of the patients being weaned from mechanical circulatory support within 48 h [44]. These results underscore the feasibility, safety, and therapeutic potential of urgent TEER in critically ill patients, particularly those with ischemic etiologies where early intervention may alter clinical trajectories.

Our findings are further corroborated by studies examining shared clinical and hemodynamic outcomes. For instance, Droppa et al. reported significant reductions in MR severity as well as improvements in left atrial pressure and cardiac index following TEER in patients with CS, without deterioration in left ventricular function—findings that align closely with our own pooled estimates [55]. Similarly, Shuvy et al. concluded that TEER is not only safe and well-tolerated in high-risk patients, but also associated with superior in-hospital and one-year mortality outcomes compared to surgical treatment, particularly in the context of post-infarction MR [56]. Collectively, these studies reinforce the position of TEER as a technically effective and physiologically beneficial intervention in select patients with severe MR and hemodynamic compromise, and support its emerging role as a less invasive yet life-saving alternative in those deemed inoperable.

The clinical implications of this meta-analysis suggest that TEER may represent a viable and effective therapeutic strategy for patients with AMR complicated by CS, particularly in those deemed unsuitable for surgical intervention due to hemodynamic instability or prohibitive operative risk [57–59]. The significant reduction in in-hospital mortality associated with TEER, coupled with high procedural success rates—as evidenced by the substantial proportion of patients achieving post procedural MR reduction to ≤ grade 2—underscores its potential utility as a minimally invasive intervention in this high-risk population. These findings support the incorporation of TEER into contemporary clinical decision-making frameworks and highlight the importance of a multidisciplinary heart team approach to facilitate optimal patient selection, procedural planning, and management in the context of AMR and CS.

### Limitations

This study has several limitations that should be considered when interpreting the findings. First, the included studies were predominantly observational in nature, with a lack of randomized controlled trials, which may introduce inherent biases such as confounding and selection bias. Specifically, patients selected for M-TEER may have been in relatively better clinical condition compared to those who did not undergo the procedure, potentially influencing the observed outcomes. Second, substantial variation existed in patient populations, procedural timing, and operator experience across studies, which may affect the generalizability of the results. Third, some outcomes, including long-term mortality and rehospitalization, were reported inconsistently or were derived from a limited number of studies, potentially reducing the precision of pooled estimates. Additionally, although we analyzed rehospitalization outcomes, the reason for rehospitalization (e.g., cardiovascular vs. non-cardiovascular causes) was not clearly specified in the majority of studies. This limitation prevented us from stratifying rehospitalization by cause, which may have provided further insight into TEER’s impact on disease-specific outcomes. Future research should prioritize well-designed studies to establish the efficacy of TEER in patients with AMR complicated by CS. Standardization in outcome reporting and patient selection criteria will be essential for enhancing comparability across studies. We also encourage future research to specifically explore outcomes stratified by emergent versus non-emergent TEER procedures, as this could provide insights into optimizing patient care. In addition, future meta-analyses should aim to conduct meta-regression and subgroup analyses to better explore potential sources of heterogeneity. Important variables to examine include the etiology of AMR, patient age and comorbidity profiles, baseline left ventricular ejection fraction, and use of adjunctive mechanical circulatory support. Moreover, cost-effectiveness analyses are warranted to inform clinical practice and health policy.

## Conclusion

This systematic review and meta-analysis demonstrates that TEER may serve as a feasible and potentially life-saving alternative for patients with AMR complicated by CS. TEER was associated with favorable procedural outcomes, including a high rate of MR reduction to ≤ grade 2 and a significantly lower in-hospital mortality compared to usual care. Despite these encouraging findings, the long-term mortality remained high, reflecting the critical nature of AMR with CS. Moreover, the current evidence base is largely derived from observational studies with methodological limitations, limiting the overall certainty of the results. Future prospective, multicenter studies and randomized controlled trials are needed to validate these findings, identify ideal candidates for TEER, and optimize timing and procedural strategies. Until more robust data are available, TEER may be considered a promising option for select high-risk patients with AMR and CS who are not suitable candidates for surgery.

## Electronic supplementary material

Below is the link to the electronic supplementary material.

Supplementary Material 1

## Data Availability

The datasets used and/or analyzed during the current study are available from the corresponding author on reasonable request.

## Abbreviations

AMR: Acute Mitral Regurgitation
CAD: Coronary Artery Disease
CI: Confidence Interval
CS: Cardiogenic Shock
GRADE: Grading of Recommendations, Assessment, Development, and Evaluations
HTN: Hypertension
IABP: Intra–Aortic Balloon Pump
JBI: Joanna Briggs Institute
LV: Left Ventricle / Ventricular
LVEF: Left Ventricular Ejection Fraction
MACE: Major Adverse Cardiovascular Events
mPAP: Mean Pulmonary Artery Pressure
MR: Mitral Regurgitation
OR: Odds Ratio
PRISMA: Preferred Reporting Items for Systematic Reviews and Meta–Analyses
PROSPERO: International Prospective Register of Systematic Reviews
REML: Restricted Maximum Likelihood
SCAI: Society for Cardiovascular Angiography and Interventions
STS: Society of Thoracic Surgeons (Risk Score)
TEER: Transcatheter Edge–to–Edge Repair
TMVR: Transcatheter Mitral Valve Repair
