# Supplementary material for: Clinical outcomes of transcatheter edge-to-edge repair in patients with acute mitral regurgitation complicated by cardiogenic shock: a systematic review and meta-analysis
Source: BMC Cardiovasc Disord. 2025 May 19;25:380. doi: 10.1186/s12872-025-04844-z (PMC12087214; doi:10.1186/s12872-025-04844-z)
Supplement: Supplementary file 1 — Supplementary Material 1 [file 12872_2025_4844_MOESM1_ESM.docx]

Table S1: Search Strategy of included databases

| Database | Search Strategy |
| --- | --- |
| PubMed | ("mitral valve transcatheter edge-to-edge repair" OR "M-TEER" OR "MTEER" OR "small mitral valve" OR "transcatheter mitral valve repair" OR "TMVr" OR "edge-to-edge technique" OR "edge-to-edge" OR "mitra clip" OR "mitraclip") AND ("Mitral Valve Insufficiency"[MeSH]) AND ("Shock, Cardiogenic"[MeSH]) |
| Embase | ('mitral valve transcatheter edge-to-edge repair':ti,ab OR 'm-teer':ti,ab OR 'mteer':ti,ab OR 'small mitral valve':ti,ab OR 'transcatheter mitral valve repair':ti,ab OR 'tmvr':ti,ab OR 'edge-to-edge technique':ti,ab OR 'edge-to-edge':ti,ab OR 'mitra clip':ti,ab OR 'mitraclip':ti,ab) AND ('mitral regurgitation'/exp OR 'mitral valve insufficiency':ti,ab OR 'insufficiency, mitral valve':ti,ab OR 'valve insufficiency, mitral':ti,ab OR 'mitral incompetence':ti,ab OR 'incompetence, mitral':ti,ab OR 'mitral insufficiency':ti,ab OR 'insufficiency, mitral':ti,ab OR 'mitral regurgitation':ti,ab OR 'regurgitation, mitral':ti,ab OR 'mitral valve incompetence':ti,ab OR 'incompetence, mitral valve':ti,ab OR 'valve incompetence, mitral':ti,ab OR 'mitral valve regurgitation':ti,ab OR 'regurgitation, mitral valve':ti,ab OR 'valve regurgitation, mitral':ti,ab) AND ('cardiogenic shock'/exp OR 'shock, cardiogenic':ti,ab OR 'cardiac shock':ti,ab OR 'circulatory shock':ti,ab OR 'post-infarction cardiogenic shock':ti,ab OR 'refractory cardiogenic shock':ti,ab OR 'acute cardiogenic shock':ti,ab OR 'low cardiac output syndrome':ti,ab) |
| Scopus | TITLE-ABS-KEY ( "mitral valve transcatheter edge-to-edge repair" OR "M-TEER" OR "MTEER" OR "small mitral valve" OR "transcatheter mitral valve repair" OR "TMVr" OR "edge-to-edge technique" OR "edge-to-edge" OR "mitra clip" OR "mitraclip" ) AND TITLE-ABS-KEY ( "Mitral Valve Insufficiency" OR "Insufficiency, Mitral Valve" OR "Valve Insufficiency, Mitral" OR "Mitral Incompetence" OR "Incompetence, Mitral" OR "Mitral Insufficiency" OR "Insufficiency, Mitral" OR "Mitral Regurgitation" OR "Regurgitation, Mitral" OR "Mitral Valve Incompetence" OR "Incompetence, Mitral Valve" OR "Valve Incompetence, Mitral" OR "Mitral Valve Regurgitation" OR "Regurgitation, Mitral Valve" OR "Valve Regurgitation, Mitral" ) AND TITLE-ABS-KEY ( "Cardiogenic Shock" OR "Shock, Cardiogenic" OR "Cardiac shock" OR "Circulatory shock" OR "Post-infarction cardiogenic shock" OR "Refractory cardiogenic shock" OR "Acute cardiogenic shock" OR "Low cardiac output syndrome" ) |
| Web | (("mitral valve transcatheter edge-to-edge repair" OR "M-TEER" OR "MTEER" OR "small mitral valve" OR "transcatheter mitral valve repair" OR "TMVr" OR "edge-to-edge technique" OR "edge-to-edge" OR "mitra clip" OR "mitraclip") And ("Mitral Valve Insufficiency" OR "Insufficiency, Mitral Valve" OR "Valve Insufficiency, Mitral" OR "Mitral Incompetence" OR "Incompetence, Mitral" OR "Mitral Insufficiency" OR "Insufficiency, Mitral" OR "Mitral Regurgitation" OR "Regurgitation, Mitral" OR "Mitral Valve Incompetence" OR "Incompetence, Mitral Valve" OR "Valve Incompetence, Mitral" OR "Mitral Valve Regurgitation" OR "Regurgitation, Mitral Valve" OR "Valve Regurgitation, Mitral") And ("Cardiogenic Shock" OR "Shock, Cardiogenic" OR "Cardiac shock" OR "Circulatory shock" OR "Post-infarction cardiogenic shock" OR "Refractory cardiogenic shock" OR "Acute cardiogenic shock" OR "Low cardiac output syndrome")) |

**Table S2**: Quality of included studies

| Number of studies | Author, year | Question 1 | Question 2 | Question 3 | Question 4 | Question 5 | Question 6 | Question 7 | Question 8 | Question 9 | Question 10 | Question 11 | Total score |
| --- | --- | --- | --- | --- | --- | --- | --- | --- | --- | --- | --- | --- | --- |
| 1 | Kovach, 2021 | Yes | Yes | Yes | Yes | Yes | Yes | Yes | Yes | Yes | Yes | Yes | 11/11 |
| 2 | Lee, 2021 | Yes | Yes | Yes | Yes | Yes | Yes | Yes | Yes | Yes | Yes | Yes | 11/11 |
| 3 | Simard, 2022 | Yes | Yes | Yes | Yes | Yes | Unclear | Yes | Yes | Yes | Yes | Yes | 10/11 |
| 4 | Haberman, 2024 | Yes | Yes | Yes | Yes | Yes | Yes | Yes | Yes | Yes | No | Yes | 10/11 |
| 5 | Jung, 2021 | Yes | Yes | Yes | Yes | Yes | Yes | Yes | Yes | Yes | Yes | Yes | 11/11 |
| 6 | Tang, 2021 | Yes | Yes | Yes | Yes | Yes | Yes | Yes | Yes | Yes | Yes | Yes | 11/11 |
| 7 | Adamo, 2017 | Yes | Yes | Yes | Yes | Yes | Yes | Yes | Yes | Yes | Yes | - | 10/10 |
| 8 | Makmal, 2024 | Yes | Yes | Yes | Yes | Yes | Yes | Yes | Yes | No | Unclear | Yes | 9/11 |
| 9 | Aldrugh, 2021 | Yes | Yes | Yes | Yes | Yes | Yes | Yes | Yes | Yes | Yes | Yes | 11/11 |
| 10 | Estevez-Loureiro, 2021 | Yes | Yes | Yes | Yes | Yes | Yes | Yes | No | Yes | Yes | Yes | 10/11 |
| 11 | So, 2022 | Yes | Yes | Yes | Yes | Yes | Yes | Yes | Yes | Yes | Yes | Yes | 11/11 |
| 12 | Falasconi, 2021 | Yes | Yes | Yes | Yes | Yes | Yes | Yes | Yes | Yes | Yes | Yes | 11/11 |
| 13 | Flint, 2019 | Yes | Yes | Yes | Yes | Yes | Yes | Yes | Yes | Unclear | Yes | Yes | 10/11 |
| 14 | Perel, 2022 | Yes | Yes | Yes | Yes | Yes | Yes | Yes | Yes | Yes | Yes | Yes | 11/11 |
| 15 | Taramasso, 2019 | Yes | Yes | Yes | Yes | Yes | Yes | Yes | Yes | Yes | Yes | Yes | 11/11 |
| 16 | Rizik, 2019 | Yes | Yes | Yes | Yes | Yes | Yes | Yes | Yes | Yes | Yes | - | 10/10 |
| 17 | Garcia, 2020 | Yes | Yes | Yes | Yes | Yes | Yes | Yes | Yes | Yes | Yes | Yes | 11/11 |
| 18 | Chitturi, 2020 | Yes | Yes | Yes | Yes | Yes | Yes | Yes | Yes | Yes | Yes | - | 10/10 |
| 19 | Vandenbriele, 2021 | Yes | Yes | Yes | Yes | Yes | Yes | Yes | Yes | Yes | Yes | - | 10/10 |
| 20 | Tanaka, 2022 | Yes | Yes | Yes | Yes | Yes | Yes | Yes | Yes | No | Yes | - | 9/10 |
| 21 | Ahmed, 2023 | Yes | Yes | Yes | No | No | Yes | Yes | Yes | No | Yes | - | 7/10 |
